# A dual role for microglia in promoting tissue inhibitor of metalloproteinase (TIMP) expression in glial cells in response to neuroinflammatory stimuli

**DOI:** 10.1186/1742-2094-8-61

**Published:** 2011-06-01

**Authors:** Jennifer V Welser-Alves, Stephen J Crocker, Richard Milner

**Affiliations:** 1 Department of Molecular and Experimental Medicine, The Scripps Research Institute, 10550 North Torrey Pines Road, La Jolla, CA 92037, USA; 2 Department of Neuroscience, University of Connecticut Health Center, Farmington, CT, USA

## Abstract

**Background:**

By neutralizing the effect of the matrix metalloproteinases (MMPs), the tissue inhibitors of matrix metalloproteinases (TIMPs) play a critical role in maintaining tissue proteolysis in balance. As the major reactive glial cell types in the central nervous system (CNS), microglia and astrocytes play fundamental roles in mediating tissue breakdown and repair. As such, it is important to define the TIMP expression profile in these cells, as well as the mechanisms of regulation by neuroinflammatory stimuli.

**Methods:**

Primary mixed glial cultures (MGC), pure microglia, and pure astrocytes were used in this study. To study astrocytes, we employed a recently described pure astrocyte culture system, which has the major advantage of totally lacking microglia. The three different types of culture were treated with lipopolysaccharide (LPS) or individual cytokines, and cell culture supernatants assayed for TIMP-1 or TIMP-2 protein expression by western blot.

**Results:**

LPS induced TIMP-1 expression in MGC, but not in pure astrocyte or microglial cultures. When pure astrocytes were treated with the cytokines IL-1β, IFN-γ, TNF or TGF-β1, only IL-1β induced TIMP-1 expression. Significantly, astrocyte TIMP-1 expression was restored in LPS-treated astrocyte cultures after the addition of microglia, or conditioned medium taken from LPS-activated microglia (MG-CM). Furthermore, this effect was lost after depletion of IL-1β from MG-CM. By contrast, TIMP-2 was constitutively expressed by astrocytes, whereas microglia expressed TIMP-2 only after exposure to serum.

**Conclusions:**

Taken together, these results demonstrate an important concept in glial interactions, by showing that microglia play a central role in regulating glial cell expression of TIMPs, and identify microglial IL-1β as playing a key role in mediating microglial-astrocyte communication.

## Background

By inhibiting the matrix metalloproteinases (MMPs), the tissue inhibitors of metalloproteinases (TIMPs) play an important role in maintaining proteolysis in balance [1-3]. In addition to this role, increasing evidence suggests that TIMPs also function in a MMP-independent manner, to regulate different aspects of cell behavior, including cell survival, cell proliferation, and the regulation of angiogenesis (reviewed in [4]).

Within the central nervous system (CNS), MMPs and TIMPs play important roles, both during development, and during the pathogenesis and remodeling that follows CNS damage [5-8]. The two major reactive glial cell types in the CNS are microglia and astrocytes. In light of the major role these cells play during CNS disease and repair, it is a high priority to define which MMPs and TIMPs are expressed by these two glial cell types, and understand how this expression is regulated during disease pathogenesis. To shed some light on this, we previously examined TIMP expression at the mRNA level by RNase protection assay in primary glial cultures. This showed that while TIMP-2 is constitutively expressed, both in astrocyte-enriched cultures and microglia, TIMP-1 is much more restricted in its expression, being expressed in astrocyte-enriched cultures only after stimulation by IL-1β or lipopolysaccharide (LPS) [9]. In these previous studies, we took the currently acceptable standard approach of using mixed glial cultures (MGC) to represent astrocytes. Importantly though, as MGC contain microglia in addition to astrocytes, it was not possible to determine whether LPS and IL-1β exert a direct effect on astrocyte TIMP-1 production, or whether this effect requires the production of microglial intermediate factors. Indeed, in light of the finding that astrocytes express only marginal levels of the LPS receptor, TLR-4 [10], it seemed more likely that the influence of LPS was mediated via microglial factors. Based on these observations, we proposed the hypothesis that LPS promotes astrocyte TIMP-1 expression through an indirect mechanism, in which LPS induces microglia to secrete a soluble factor (possibly IL-1β), which then stimulates astrocyte TIMP-1 expression. These previous findings indicate the existence of an important interplay between microglia and astrocytes in their regulation of gene expression. As it is becoming clear that astrocytes play important roles in regulating many aspects of CNS function, including myelination [11] and microglial reactivity [12], identifying how other CNS cell types influence astrocyte functions becomes an important priority.

To gain an improved understanding of the significance of microglial-astrocyte interactions in these responses, we recently developed and characterized a novel method of obtaining pure astrocytes, in which neurospheres containing neural stem cells (NSC) are differentiated into astrocytes in the presence of 10% fetal bovine serum (FBS) [13]. This approach yields pure astrocyte cultures, totally devoid of microglia, thus permitting an analysis of astrocyte protein expression, not complicated by the presence of contaminating microglia. We have already used this system to good effect to demonstrate that astrocytes and microglia show distinct, non-overlapping expression profiles of MMPs. Using this improved method, we found that in contrast to MGC, pure astrocyte cultures express a limited repertoire of MMPs, quite distinct from the microglial pattern, highlighted by the finding that microglia, not astrocytes express MMP-9 [13]. This study also showed that in contrast to MGC or microglia, pure astrocytes appear to be unresponsive to LPS, showing no alterations in MMP expression patterns, consistent with the finding that astrocytes express only low levels of the LPS receptor, TLR-4 [10].

The purpose of the current study was to employ the improved pure astrocyte culture system, and together with pure microglial cultures, define the expression of TIMP-1 and TIMP-2 in astrocytes or microglia at the protein level, and examine how this is regulated by cytokines and LPS. Then, test our hypothesis that the effect of LPS on astrocyte TIMP-1 expression is mediated via a microglial-secreted factor, and if so, identify the molecular mechanism responsible.

## Methods

### Animals

The studies described have been reviewed and approved by The Scripps Research Institute Institutional Animal Care and Use Committee. All cell cultures were obtained from C57Bl/6 mice which were maintained under pathogen-free conditions in the closed breeding colony of The Scripps Research Institute (TSRI).

### Cell culture

Mixed glial cultures (MGC) were prepared from 0-2 day old C57Bl/6 mouse pups, as previously described [14], and maintained in poly-D-lysine coated T75 flasks in DMEM containing 10% fetal bovine serum (FBS) (all from Sigma, St. Louis, MO). After 7-10 days, MGC were shaken for 30 minutes and the supernatant containing detached microglia was collected. Microglia were counted by hemocytometer, re-suspended in the shake-off medium, and plated at a density of 2 × 10^5 ^cells/well in poly-D-lysine coated 6-well plates (Nunc, Naperville, IL). Microglial purity was > 99% as determined by Mac-1 in flow cytometry. After MGC were harvested of microglial cells, the MGC were passaged into poly-D-lysine coated 6-well plates and maintained in DMEM containing 10% FBS. Pure astrocyte cultures were prepared as previously described [13], by plating neurospheres into poly-D-lysine coated 6-well plates and maintained in DMEM containing 10% FBS. Astrocyte purity of these neurosphere-derived cultures was > 99% as determined by GFAP immunocytochemistry.

### Treatment of glial cultures with LPS and cytokines

After microglia were shaken off MGC, they were allowed to attach overnight, before the medium was changed next day to serum-free DMEM containing N1-supplement, L-glutamine and penicillin/streptomycin (all from Sigma). In experiments designed to evaluate the role of microglia in promoting TIMP-1 expression in astrocytes, 0.5 × 10^5 ^microglia were added per well of astrocytes. MGC and pure astrocyte cultures were maintained in DMEM containing 10% FBS until confluency, then switched to the same N1-supplemented serum-free medium. Upon switching to serum-free medium, all three types of culture received either no treatment, lipopolysaccharide (LPS, serotype: E. Coli 0111.B4, 1 μg/ml, Sigma), IFN-γ (10 U/ml, R&D, Minneapolis, MN), IL-1β (10 ng/ml, Peprotech, Rocky Hill, NJ), TNF-α (20 ng/ml, Genentech, San Francisco, CA), or TGF-β1 (20 ng/ml, R&D). After 2 days treatment, cell culture supernatants were collected for analysis. LPS-activated microglial conditioned medium (MG-CM) was produced and collected the same way, and filtered through a 0.22 μm filter before addition to pure astrocyte cultures.

### Antibodies

The goat polyclonal antibodies specific for TIMP-1 (AF-980), TIMP-2 (AF-971), and IL-1β (AF-401-NA) were obtained from R&D Systems. Sources of other antibodies include: rabbit anti-goat polyclonal antibody (Vector Labs, Burlingame, CA), mouse anti-GFAP-Cy3 (Sigma, cat. no. C9205, clone G-A-5), rat anti-Mac-1 antibody (BD Pharmingen, La Jolla, CA, cat. no. 553310, clone M1/70), anti-rat-Alexafluor 488 (Invitrogen, Carlsbad, CA), and anti-goat-HRP (Pierce, Rockford, IL).

### Immunocytochemistry

To investigate the purity of the different glial culture systems, cells were cultured on fibronectin coated glass coverslips in DMEM containing 10% FBS. Cells were washed in PBS and fixed in acid/alcohol (95:5) at -20°C for 10 minutes, before being washed extensively and blocked in 5% normal goat serum (NGS) in PBS for 30 minutes. Cells were then incubated with a Mac-1 monoclonal antibody for 1 hour at room temperature, washed and then incubated with anti-rat-Alexafluor 488 for 30 minutes at room temperature. After washing, cells were incubated with a mouse anti-GFAP-Cy3 conjugated antibody for 1 hour, washed and then incubated with the nuclear Hoechst stain (Sigma) for 10 minutes. Coverslips were then washed and mounted in aquamount (Polysciences, Warrington, PA).

### Immunodepletion experiments

LPS-activated MG-CM was immunodepleted of IL-1β by six successive rounds of immunoprecipitation. Briefly, the cell culture supernatants were first pre-cleared for one hour with 30 μl of protein A sepharose beads per ml of cell lysate. This was followed by six consecutive immunoprecipitations, in which 4 μl of goat anti-IL-1β and 4 μl of rabbit anti-goat antibody were added to a tube containing 1 ml MG-CM and 30 μl beads. Each ID was performed for a minimum period of 4 hours, all at 4°C on a rotating platform. For control, the same MG-CM also underwent a mock immunodepletion containing 4 μl of normal goat serum and 4 μl of rabbit anti-goat antibody. After the last ID, the supernatants were sterile filtered through a 0.22 μm filter before addition to pure astrocyte cultures.

### Western blotting

Cell culture supernatants were boiled in reducing sample buffer for 5 minutes before being analysed by 12% SDS-PAGE (Invitrogen) under reduced conditions. 10 μl of cell culture supernatants were electro-blotted for 1 hour onto nitrocellulose membranes (Invitrogen), blocked overnight in 3% non-fat milk in TBS containing 0.1% Tween-20 (Sigma) and probed with the anti-TIMP or anti-IL-1β antibodies for one hour, before being washed, then probed with anti-goat-HRP conjugate (Pierce) for one hour, and then washed extensively. Protein bands were visualised with the SuperSignal WestFemto ECL detection system (Pierce) according to the manufacturers' instructions. Band intensity was quantified using the BioRad Imaging system. Results represent the mean ± SEM of 4 experiments, with each sample examined in triplicate within each experiment. Statistical significance was assessed by using Student's t test, in which p < 0.05 was defined as statistically significant.

## Results

### LPS induces TIMP-1 expression in mixed glial cultures, but not pure cultures of astrocytes or microglia

**Figure 1:**
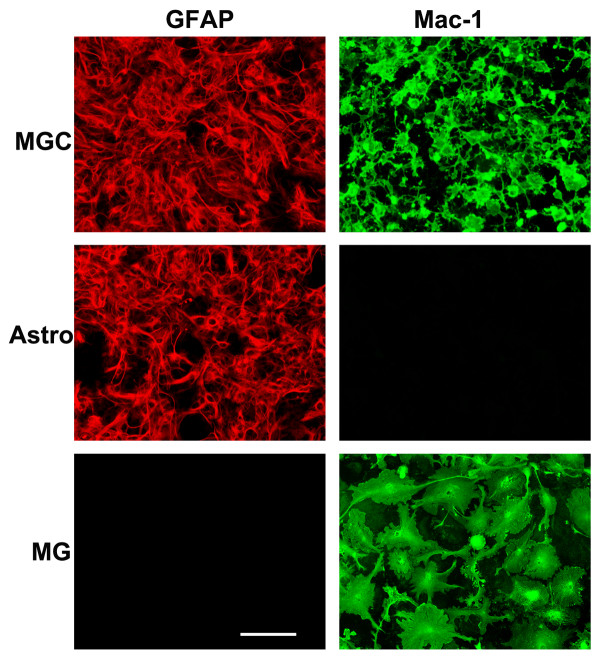
**Defining the cell purity of the three different glial cell culture systems**. Mixed glial cultures (MGC), pure astrocytes (Astro) and pure microglial cultures (MG) were established as described in Materials and Methods, and then analyzed by immunofluorescence for expression of the astrocyte marker GFAP (Cy3, red) or the microglial marker Mac-1 (AlexaFluor 488, green). Scale bar = 50 μm. Note that MGC contain both astrocytes and microglia. In contrast, neurosphere-derived pure astrocyte cultures contain only astrocytes with no microglia, whereas pure microglial cultures contain only microglia.

**Figure 2:**
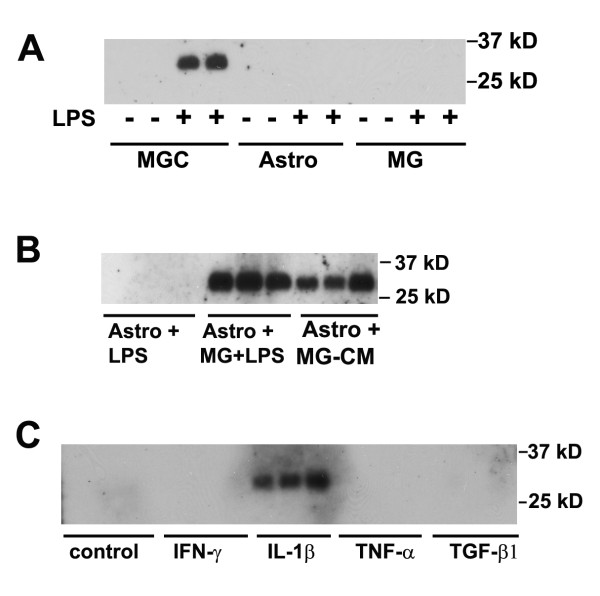
**Analysis of glial cell TIMP-1 expression in response to cytokines and LPS**. TIMP-1 expression was examined by western blot of cell culture supernatants, as described in Materials and Methods. A. The influence of LPS on TIMP-1 expression in the three different types of glial culture. MGC, astrocytes, and microglial cultures were treated with LPS for 2 days, then cell culture supernatants examined for TIMP-1 expression. Note that cultures not treated with LPS did not express TIMP-1, and TIMP-1 protein (~28 kD band) was induced only in MGC following LPS stimulation. B. TIMP-1 induction in astrocytes in the presence of activated microglia or MG-CM. Pure astrocyte cultures received either: LPS, microglia + LPS, or LPS-activated MG-CM. Note that the presence of LPS-activated microglia or LPS-activated MG-CM strongly induced TIMP-1 expression in astrocytes. C. The influence of cytokines on TIMP-1 expression by astrocytes. Pure astrocyte cultures were treated with a panel of different cytokines for 2 days, and the supernatants analyzed for TIMP-1 expression. Note that of the cytokines tested, only IL-1β stimulated TIMP-1 expression in astrocytes.

In a previous study, we showed that LPS and IL-1β promote TIMP-1 expression in mixed glial cultures (MGC), containing astrocytes and microglia, but not in pure microglial cultures [9]. From this we hypothesized that LPS promotes microglia to express soluble factors (possibly IL-1β and/or other factors), which then stimulate TIMP-1 expression in astrocytes. As the MGC system contains microglia in addition to astrocytes, it was not possible to directly test this idea using the MGC system. However, with the advent of our recently described pure astrocyte culture system, which is entirely free of microglia [13], it now becomes possible to perform a molecular dissection of the signals mediated between microglia and astrocytes. The three different glial culture systems are shown in Figure 1, with astrocytes and microglia identified by GFAP and Mac-1 positivity, respectively. MGC contained both astrocytes and microglia, pure astrocyte cultures were entirely devoid of microglia, while pure microglia cultures contained no astrocytes. All experiments were performed under serum-free conditions, and TIMP protein expression examined by western blot of cell culture supernatants. In the first experiment, we examined TIMP-1 expression in three different types of culture: MGC, pure astrocytes, or pure microglia, with or without the addition of LPS. After 2 days incubation, TIMP-1 expression was examined. As shown in Figure 2A, TIMP-1 expression was never seen in any of the three types of culture untreated with LPS, and of the LPS-treated cells, only the MGC expressed TIMP-1 protein (running at the predicted molecular weight of 28 kD). This result confirmed our previous observation (performed at the mRNA level), showing that MGC express TIMP-1 in response to LPS stimulation, but microglia do not, and also make the important point that pure astrocyte cultures are unresponsive to LPS stimulation. As MGC contain a mix of astrocytes and microglia, these results suggest that microglia are required to mediate the influence of LPS on TIMP-1 expression in astrocytes.

### Pure astrocytes express TIMP-1 in the presence of LPS-activated microglia conditioned medium

To directly test whether microglia or their secreted factors influence astrocyte TIMP-1 expression, we cultured pure astrocytes either alone, in the presence of added microglial cells, or in the presence of microglia conditioned medium (MG-CM) previously stimulated by LPS, all cultures being stimulated with LPS. As shown in Figure 2B, whereas pure astrocyte cultures treated with LPS expressed no TIMP-1, the addition of microglial cells or LPS-activated MG-CM promoted robust TIMP-1 expression. This result confirms that microglia are required to mediate the effect of LPS on astrocyte TIMP-1 expression, and furthermore shows that the effect is mediated via a microglial soluble factor. Based on our previous finding that of a panel of different cytokines tested, only IL-1β elevated TIMP-1 mRNA levels in MGC [9], we investigated a number of different cytokines for their effect to stimulate TIMP-1 expression in pure astrocyte cultures. Pure astrocytes were treated with IL-1β, IFN-γ, TNF or TGF-β1, their supernatants collected after 2 days, and examined for TIMP-1 expression (see Figure 2C). Consistent with our previous mRNA data, this demonstrated that IL-1β was the only factor that promoted TIMP-1 in pure astrocytes, and furthermore, showed that none of the cytokines stimulated TIMP-1 expression in microglial cultures (not shown).

### IL-1β is the dominant microglial factor responsible for mediating astrocyte TIMP-1 expression in response to LPS

**Figure 3:**
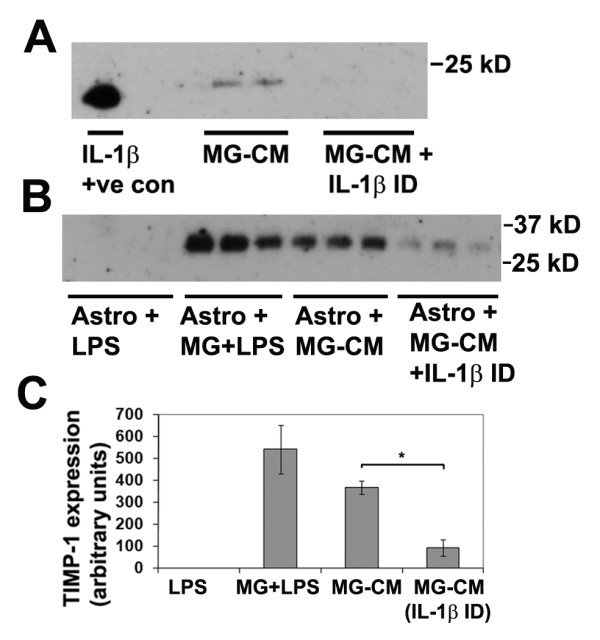
**Examining the role of microglia-derived IL-1β in promoting astrocyte TIMP-1 expression**. A. Determination of IL-1β in LPS-activated MG-CM. Microglial cultures were treated with LPS for 2 days, then subject to either six rounds of IL-1β immunodepletion, or mock depletion, as described in Materials and Methods, and samples analyzed for the presence of IL-1β. Note that IL-1β was detected in the LPS-activated MG-CM before, but not after the IL-1β immunodepletion, demonstrating the effectiveness of the immunodepletion strategy. B and C. The effect of removing IL-1β from LPS-activated MG-CM on astrocyte TIMP-1 production. Pure astrocyte cultures received either: LPS, microglia + LPS, LPS-activated MG-CM (mock depleted) or LPS-activated MG-CM depleted of IL-1β. Note that the presence of LPS-activated microglia or LPS-activated MG-CM strongly induced TIMP-1 expression in astrocytes, but this effect was markedly attenuated after IL-1β removal from the MG-CM. * p < 0.02.

Having confirmed that IL-1β has a direct effect on TIMP-1 induction in astrocytes, we next addressed the question: is IL-1β the major microglial factor that mediates the LPS-induction of astrocyte TIMP-1 expression? To answer this question, we took LPS-activated MG-CM and removed all the available IL-1β by performing six successive rounds of immunodepletion with a polyclonal anti-IL-1β antibody. Following this procedure, we first established that the immunodepletion had worked, by examining IL-1β levels in the MG-CM, before and after this procedure. As shown in Figure 3A, IL-1β was detected in the MG-CM prior to, but not after IL-1β immunodepletion, demonstrating that this procedure effectively removed all IL-1β from the MG-CM. Next, we examined TIMP-1 expression in pure astrocyte cultures treated with LPS-activated MG-CM, in which the MG-CM had either been mock-depleted (control) or really depleted of IL-1β. As shown in Figures 3B and 3C, this demonstrated that removal of IL-1β from the LPS-activated MG-CM significantly reduced astrocyte TIMP-1 expression (from 365.6 ± 31.0 under control conditions to 91.2 ± 36.3 units after IL-1β immunodepletion, p < 0.02) demonstrating that IL-1β is the dominant microglial factor responsible for mediating astrocyte TIMP-1 expression in response to LPS

### Under serum-free conditions, astrocytes but not microglia constitutively express TIMP-2

**Figure 4:**
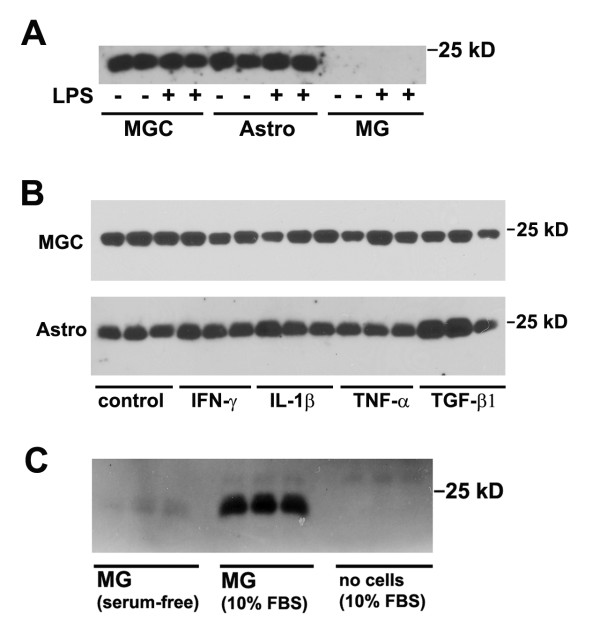
**Analysis of glial cell TIMP-2 expression in response to cytokines and LPS**. TIMP-2 expression was examined by western blot of cell culture supernatants, as described in Materials and Methods. A. The influence of LPS on TIMP-1 expression in the three different types of glial culture. MGC, astrocytes, and microglial cultures were treated with LPS for 2 days, then cell culture supernatants examined for TIMP-2 expression. Note that MGC and pure astrocytes constitutively expressed TIMP-2 (~22 kD) band, and this expression was not enhanced by LPS stimulation. In contrast, microglial cultures did not express TIMP-2. B. The influence of cytokines on TIMP-2 expression by MGC and pure astrocytes. MGC and pure astrocyte cultures were treated with a panel of different cytokines for 2 days, and the supernatants analyzed for TIMP-1 expression. Note that both MGC and pure astrocytes constitutively expressed TIMP-2, and this expression level was not significantly affected by any of the cytokines tested. C. The influence of serum on microglial TIMP-2 expression. Pure microglial cultures were grown either in serum-free medium or in medium containing 10% FBS. After 2 days, the cell culture supernatants were analyzed for TIMP-2 expression. Note that the presence of serum strongly induced TIMP-2 expression in microglial cells.

In parallel with our analysis of TIMP-1 expression in astrocyte and microglial cultures, we also examined the expression of TIMP-2 in these cultures. In contrast to TIMP-1 expression, this demonstrated that TIMP-2 was expressed at high levels both by MGC and pure astrocytes, but not microglia, and furthermore, not affected by LPS (see Figure 4A). This confirms that astrocytes constitutively express TIMP-2. To examine whether TIMP-2 expression was regulated by cytokines, we examined the same panel of cytokines, as was used in the TIMP-1 experiment. This showed that TIMP-2 expression in MGC or in pure astrocyte cultures was not affected by any of the cytokines tested (see Figure 4B). Furthermore, none of the cytokines tested induced TIMP-2 expression in microglia (not shown).

### Serum induces TIMP-2 expression in microglial cultures

Our current study failed to show any TIMP-2 expression in microglia. This was unexpected in light of our previous findings using an RNase protection assay approach, which demonstrated TIMP-2 expression, both in MGC and in microglia. In fact, this data showed that TIMP-2 was the only TIMP expressed by microglia [9]. In trying to pinpoint the reason for this discrepancy, we noted that in the first study, the cell cultures were maintained in serum-containing medium (10% FBS), while in the current study, cells were maintained under serum-free conditions. To test whether serum accounted for the difference between the two studies in microglial TIMP-2 expression, we cultured microglia in either serum-free or serum-containing media for two days, then examined TIMP-2 expression by western blot. As shown in Figure 4C, in the absence of serum, microglia expressed no TIMP-2 protein, while in the presence of serum, a dramatic increase in TIMP-2 expression was observed. To exclude the possibility that the TIMP-2 protein was serum-derived, we also examined the presence of TIMP-2 in the 10% FBS-containing medium that the cells were grown in; this failed to show any TIMP-2 protein. Therefore we conclude that serum strongly induces TIMP-2 expression in microglial cells.

## Discussion

In this study we used pure cultures of astrocytes or microglia to examine the expression of TIMP-1 and TIMP-2 at the protein level. Consistent with our previous study, which examined these events at the mRNA level [9], we found that LPS induced TIMP-1 expression in astrocyte-enriched MGC (which contain microglia in addition to astrocytes), but interestingly, this effect was not seen in pure astrocyte or microglial cultures. Significantly, astrocyte TIMP-1 expression was restored in pure astrocyte cultures after the addition of microglia, or conditioned medium taken from LPS-activated microglia (MG-CM), and this effect was lost after depletion of IL-1β from MG-CM. In contrast, TIMP-2 was constitutively expressed by astrocytes, while microglia expressed TIMP-2 only after exposure to serum. Taken together, these results demonstrate an important concept in glial interactions, by showing that microglia play a central role in regulating glial cell expression of TIMPs. Most importantly, they provide direct evidence that LPS promotes astrocyte TIMP-1 expression via an indirect mechanism in which LPS stimulates microglia to secrete IL-1β, which then induces astrocyte TIMP-1 expression.

### Functions of TIMPs in the CNS

The TIMP family is comprised of four members, TIMP-1 through TIMP-4 [1,2,4]. TIMPs were initially characterized as naturally-occurring inhibitors of the MMPs that were important for the maintenance of proteolytic balance and as such play important roles in a number of developmental and physiological remodelling processes, including cell migration, wound healing, neuronal survival and angiogenesis (reviewed in [1,2,4]). The importance of the protective MMP-neutralizing effect of TIMP-1 in the CNS is best illustrated by the finding that TIMP-1 knockout mice display increased levels of MMP-9 activity, BBB breakdown and size of ischemic insult following focal cerebral ischemia [15]. In support of this, a parallel study revealed that adenoviral delivery of TIMP-1 or TIMP-2 resulted in reduced levels of neuronal death in a model of global cerebral ischemia [16]. TIMP-1 also plays an important MMP-dependent role in demyelinating disease, as illustrated by increased levels of demyelination and myelin pathology in TIMP-1 deficient mice in the mouse model of multiple sclerosis, experimental autoimmune encephalomyelitis (EAE) [17]. In addition to the well-described MMP-dependent actions, in more recent years, several studies have demonstrated that TIMPs also have a number of MMP-independent actions, that include the regulation of cell growth and cell death (reviewed in [4]). Specifically, it has been shown that TIMP-2 promotes neuronal differentiation by inhibiting cell proliferation, in an MMP-independent manner [18,19]. Exactly how these MMP-independent effects are mediated at the molecular level is still to be determined. However, studies on endothelial cells, highly relevant because of the influence of TIMPs on angiogenesis, suggest that TIMPs can bind to a variety of cell surface receptors, including the vascular endothelial growth factor (VEGF) receptor-2 [20], and the α3β1 integrin [21], raising the possibility that TIMPs signal through these specific receptors.

Recent studies have described two unexpected roles for TIMP-1 in the regulation of neural cell behaviour. First, TIMP-1 influences the growth and morphology of cortical neurons in an MMP-dependent manner; recombinant TIMP-1 reduced neurite length while dramatically increasing the size of growth cones [22]. Second, a series of studies have demonstrated that TIMP-1 plays an important role in the generation and differentiation of oligodendrocytes [23]. TIMP-1 knockout mice show reduced levels of myelin after induction of EAE [17], whereas mice that express higher levels of TIMP-1 in their CNS exhibit attenuated demyelination in EAE [24]. This is supported by the observations that TIMP-1 KO mice show delayed oligodendrocyte differentiation during development [23], and that TIMP-1 KO neural stem cells (NSC) yield significantly fewer oligodendrocytes compared to wild type, an effect that can be reversed by the addition of recombinant TIMP-1 [23].

### TIMP expression by astrocytes and microglia

In vivo studies show that TIMP expression is more strongly associated with astrocytes than microglia [25,26]. In particular, the consensus view is that TIMP-1 expression is astrocyte-specific, and this expression is highly upregulated during neuroinflammatory states involving breakdown of the blood-brain barrier and leukocyte infiltration into the CNS [27-30], suggesting that astrocyte TIMP-1 may play an important role in curtailing the influx of activated infiltrating leukocytes, by inhibiting the action of the proteolytic enzyme, MMP-9. This idea is supported by the finding that in a mouse model of focal cerebral ischemia, TIMP-1 knockout mice display increased levels of MMP-9 activity, BBB breakdown and size of ischemic insult [15]. In addition to TIMP-1, astrocyte expression of TIMP-2 has also been described in vivo following astrocyte activation after brain injury [31,32]. Interestingly, TIMP-2 expression has also been described in microglia following brain trauma [33]. These findings are in keeping with our own observations, namely that TIMP-1 appears to be astrocyte-specific, but TIMP-2 can be expressed by both astrocytes and microglia.

A small number of studies have also examined TIMP expression in pure microglial cultures, with mixed results. One study performed with PCR on human primary microglia described all four TIMP family members within microglia [34], and an ELISA study using the human microglial cell line CHME3 described both TIMP-1 and TIMP-2 present in microglia [35]. In contrast, examination of primary mouse glial cultures showed that TIMP-1 protein was only present in astrocytes, not microglia [36], and our prior study using RNAse protection assay was entirely consistent with this finding [9]. Indeed, our current finding lends further weight to this idea by showing that microglia expressed no TIMPs under serum-free conditions, but show strong induction of TIMP-2 only after exposure to serum. This finding has two important implications. First, it suggests that some of the differences apparent between the studies of microglial TIMP expression may be explained by the use of serum in microglial cultures. All of the above described studies (including our RNAse protection assay analysis) used serum-containing media, which may not be the best representation of the in vivo condition. Of course, it is also possible that species-species variation may contribute to these differences. Second, as our data clearly demonstrates that serum strongly promotes TIMP-2 expression in microglia, this implies that breakdown of the BBB will trigger microglial TIMP-2 expression, thereby limiting the destructive power of the oncoming wave of MMP-9 introduced into the breached CNS. This is remarkably similar to our previously described serum-induction of TIMP-1 expression in astrocytes [9]. Taken together, it suggests that introduction of serum into the brain induces TIMP expression in both astrocytes (TIMP-1) and microglia (TIMP-2) as part of an intrinsic damage limitation program.

One clear point to emerge from this study is that the bacterial toxin, LPS does not directly stimulate TIMP-1 expression in astrocytes; rather it mediates its effect by inducing microglia IL-1β expression, which then triggers astrocyte TIMP-1 expression. This is important because it demonstrates that microglia play a pivotal protective role in driving glial TIMP expression in two ways, first, by upregulating TIMP-2 in response to serum, and second, by facilitating astrocyte TIMP-1 expression is response to bacterial infection. It also shows that the pro-inflammatory cytokine IL-1β has two sides to its character; having not only deleterious effects in the CNS, but also providing certain important protective functions. In the current study we have shown that IL-1β is the major microglial secreted factor responsible for triggering astrocyte TIMP-1 expression. Removal of IL-1β resulted in a 75% reduction in astrocyte TIMP-1 expression. That astrocytes still showed a small TIMP-1 response might be due to the presence of another factor within the MG-CM, or alternatively, it could be due to a failure of our immunodepletion strategy to totally remove all IL-1β from the MG-CM. In fact, the latter seems more likely, as it is almost impossible to totally remove a factor, so that even a small amount of IL-1β might be capable of eliciting some degree of TIMP-1 response. If true, this suggests that neuroinflammation in IL-1β knockout mice may result in an absent astrocyte TIMP-1 response, and a potentially much stronger MMP-proteolytic effect.

## Conclusions

The aim of this study was to examine whether TIMP-1 and TIMP-2 are expressed in pure cultures of astrocytes and microglia, and then define the influence of LPS and cytokines on this expression. While LPS induced TIMP-1 expression in astrocyte-enriched MGC (which contain microglia in addition to astrocytes), this effect was not seen in pure astrocyte or microglial cultures. However, the addition of microglia, or conditioned medium taken from LPS-activated microglia (MG-CM) restored astrocyte TIMP-1 expression, and this effect was lost after depletion of IL-1β from MG-CM. By contrast, TIMP-2 was constitutively expressed by astrocytes, and not altered by any of the factors tested, while microglia expressed TIMP-2 only after exposure to serum. These results demonstrate an important concept in glial interactions, by showing that microglia play a central role in regulating glial cell expression of TIMPs, and identify microglial IL-1β as playing a key role in mediating microglial-astrocyte communication.

## Competing interests

The authors declare that they have no competing interests.

## Authors' contributions

JVW carried out the biochemical analysis and contributed to drafting the manuscript. SJC participated in the design of the study and also assisted in manuscript preparation. RM conceived of the study, prepared the cell cultures, and drafted the manuscript. All authors read and approved the final manuscript.

## References

[B1] Crocker SJ Pagenstecher A Campbell IL The TIMPs tango with MMPs and more in the central nervous system J Neurosci Res 2004 75 1 11 10.1002/jnr.10836 14689443

[B2] Gardner J Ghorpade A Tissue inhibitor of metalloproteinase (TIMP)-1: the TIMPed balance of matrix metalloproteinases in the central nervous system J Neurosci Res 2003 74 801 806 10.1002/jnr.10835 14648584 PMC3857704

[B3] Agrawal SM Lau L Yong VW MMPs in the central nervous system: where the good guys go bad Semin Cell Dev Biol 2008 19 42 51 10.1016/j.semcdb.2007.06.003 17646116

[B4] Stetler-Stevenson WG Tissue inhibitors of metalloproteinases in cell signaling: metalloproteinases-independent biological activities Sci Signal 2008 1 re6 10.1126/scisignal.127re6 18612141 PMC2493614

[B5] Candelario-Jalil E Yang Y Rosenberg GA Diverse roles of matrixmetalloproteinsases and tissue inhibitors of metalloproteinases in neuroinflammation and cerebral ischemia Neuroscience 2009 158 983 994 10.1016/j.neuroscience.2008.06.025 18621108 PMC3584171

[B6] Cunningham LA Wetzel M Rosenberg GA Multiple roles for MMPs and TIMPs in cerebral ischemia Glia 2005 50 329 339 10.1002/glia.20169 15846802

[B7] Rosell A Lo EH Multiphasic roles for matrix metalloproteinases after stroke Curr Opin Pharmacol 2008 8 82 89 10.1016/j.coph.2007.12.001 18226583

[B8] Zhao BQ Wang S Kim hY Storrie H Rosen BR Mooney DJ Wang XL Lo EH Role of matrix metalloproteinases in delayed cortical responses after stroke Nat Med 2006 12 441 445 10.1038/nm1387 16565723

[B9] Crocker SJ Milner R Pham-Mitchell N Campbell IL Cell and agonist-specific regulation of genes for matrix metalloproteinases and their tissue inhibitors by primary glial cells J Neurochem 2006 98 812 823 10.1111/j.1471-4159.2006.03927.x 16893421

[B10] Jack CS Arbour N Manusow J Montgrain V Blain M McCrea E Shapiro A Antel JP TLR signaling tailors innate immune responses in human microglia and astrocytes J Immunol 2005 175 4320 4330 16177072 10.4049/jimmunol.175.7.4320

[B11] Watkins TA Emery B Mulinyawe S Barres BA Distinct stages of myelination regulated by gamma-secretase and astrocytes in a rapidly myelinating CNS coculture system Neuron 2008 60 555 569 10.1016/j.neuron.2008.09.011 19038214 PMC2650711

[B12] Lee M Schwab C McGeer PL Astocytes are GAGAergic cells that modulate microglial activity Glia 2011 59 152 165 10.1002/glia.21087 21046567

[B13] Crocker SJ Frausto RF Whitton JL Milner R A novel method to establish microglia-free astrocyte cultures: comparison of matrix metalloproteinase expression profiles in pure cultures of astrocytes and microglia Glia 2008 56 1187 1198 10.1002/glia.20689 18449943 PMC2776034

[B14] Milner R Hung S Wang X Berg G Spatz M del Zoppo G Responses of endothelial cell and astrocyte matrix-integrin receptors to ischemia mimic those observed in the neurovascular unit Stroke 2008 39 191 197 10.1161/STROKEAHA.107.486134 18032737 PMC2588548

[B15] Fujimoto M Takagi Y Aoki T Harase M Marumo T Gomi M Nishimura M Kataoka H Hashimoto N Nozaki K Tissue inhibitor of metalloproteinases protect blood-btain barrier disruption in focal cerebral ischemia J Cereb Blood Flow Metab 2008 28 1674 1685 10.1038/jcbfm.2008.59 18560439

[B16] Magnoni S Baker A Thomson S Jordan G George SJ McColl BW McCulloch J Horsburgh K Neuroprotective effect of adenoviral-mediated gene transfer of TIMP-1 and -2 in ischemic brain injury Gene Ther 2007 14 621 625 10.1038/sj.gt.3302894 17235293

[B17] Crocker SJ Whitmire JK Frausto RF Chertboonmuang P Soloway PD Whitton JL Campbell IL Persistent macrophage/microglial activation and myelin disruption after experimental autoimmune encephalomyelitis in tissue inhibitor of metalloproteinase-1-deficient mice Am J Pathol 2006 169 2104 2116 10.2353/ajpath.2006.060626 17148673 PMC1762490

[B18] Perez-Martinez L Jaworski DM Tissue inhibitor of metalloproteinase-2 promotes neuronal differentiation by acting as an anti-mitogenic signal J Neurosci 2005 25 4917 4929 10.1523/JNEUROSCI.5066-04.2005 15901773 PMC1282460

[B19] Jaworski DM Perez-Martinez L Tissue inhibitor of metalloproteinase-2 (TIMP-2) expression is regulated by multiple neural differentiation signals J Neurochem 2006 98 234 247 10.1111/j.1471-4159.2006.03855.x 16805810 PMC2987570

[B20] Qi JH Ebrahem Q Moore N Murphy G Claesson-Welsh L Bond M Baker A Anand-Apte B A novel function for tissue inhibitor of metalloproteinases-3 (TIMP-3): inhbition of angiogenesis by blockage of VEGF binding to VEGF receptor-2 Nat Med 2003 9 407 415 10.1038/nm846 12652295

[B21] Seo DW Li H Guedez L Wingfield PT Diaz T Salloum R Wei BY Stetler-Stevenson WG TIMP-2 mediated inhbition of angiogenesis: an MMP-independent mechanism Cell 2003 114 171 180 10.1016/S0092-8674(03)00551-8 12887919

[B22] Ould-yahoui A Tremblay E Sbai O Ferhat L Bernard A Charrat E Gueye Y Lim NH Brew K Risso JJ Dive V Khrestchatisky M Rivera S A new role for TIMP-1 in modulating neurite outgrowth and morphology of cortical neurons PLoS One 2009 4 e8289 10.1371/journal.pone.0008289 20011518 PMC2788270

[B23] Moore C Milner R Nishiyama A Frausto F Serwanski D Pagarigan R Whitton JL Miller R Crocker SJ Astrocytic TIMP-1 promotes oligodendrocyte differentiation and enhances CNS myelination J Neurosci 2011 31 6247 54 10.1523/JNEUROSCI.5474-10.2011 21508247 PMC3090636

[B24] Althoff GE Wolfer DP Timmesfeld N Kanzler B Schrewe H Pagenstecher A Long-term expression of tissue inhibitor of matrix metalloproteinase-1 in the murine central nervous system does not alter the morphological and behavioral phenotype but alleviates the course of experimental allergic encephalomyelitis Am J Pathol 2010 177 840 853 10.2353/ajpath.2010.090918 20558576 PMC2913353

[B25] Rivera S Ogier C Jourquin J Timsit S Szklarcyzk AW Miller KM Gearing AJ Kaczmarek L Khrestchatisky M Gelatinase B and TIMP-1 are regulated in a cell- and time-dependent manner in association with neuronal death and glial reactivity after global forebrain ischemia Eur J Neurosci 2002 15 19 32 10.1046/j.0953-816x.2001.01838.x 11860503

[B26] Bugno M Witek B Bereta J Bereta M Edwards DR Kordula T Reprogramming of TIMP-1 and TIMP-3 expression profiles in brain microvascular endothelial cells and astrocytes in response to proinflammatory cytokines FEBS Lett 1999 448 9 14 10.1016/S0014-5793(99)00323-3 10217399

[B27] Pagenstecher A Lassmann S Carson MJ Kincaid CL Stalder AK Campbell IL Astrocyte-targeted expression of IL-12 induces active cellular immune responses in the central nervous system and modulates experimental allergic encephalomyelitis J Immunol 2000 164 4481 4492 10779748 10.4049/jimmunol.164.9.4481

[B28] Pagenstecher A Stalder AK Kincaid CL Shapiro L Campbell IL Differential expression of matrix metalloproteinase and tissue inhibitor of matrix metalloproteinase genes in the mouse central nervous system in normal and inflammatory states Am J Pathol 1998 152 729 741 9502415 PMC1858390

[B29] Teesalu T Hinkkanen AE Vaheri A Coordinated induction of extracellular proteolysis systems during experimental autoimmune encephalomyelitis in mice Am J Pathol 2001 159 2227 2237 10.1016/S0002-9440(10)63073-8 11733372 PMC1850601

[B30] Clark RT Philip-Nance J Noor S Wilson EH T-cell production of matrix metalloproteinases and inhibition of parasite clearance by TIMP-1 during chronic Toxoplasma infection in the brain ASN Neuro 2011 3 pii: e00049 21434872 10.1042/AN20100027 PMC3024837

[B31] Muir EM Adcock KH Morgenstern DA Clayton R von Stillfried N Rhodes K ellis C Fawcett JW Rogers JH Matrix metallproteinases and their inhbitors are produced by overlapping populations of activated astrocytes Brain Res Mol Brain Res 2002 100 103 117 12008026 10.1016/s0169-328x(02)00132-8

[B32] Agapova OA Ricard CS Salvador-Silva M Hernandez MR Expression of matrix metalloproteinases and tissue inhibitors of metalloproteinases in human optic nerve head astrocytes Glia 2001 33 205 216 10.1002/1098-1136(200103)33:3<205::AID-GLIA1019>3.0.CO;2-D 11241738

[B33] Jaworski DM Differential regulation of tissue inhibitor of metalloproteinase mRNA expression in response to intracranial injury Glia 2000 30 199 208 10.1002/(SICI)1098-1136(200004)30:2<199::AID-GLIA9>3.0.CO;2-# 10719361

[B34] Nuttall RK Silva C Hader W Bar-Or A Patel KD Edwards DR Yong VW metalloproteinases are enriched in microglia compared with leukocytes and they regulate cytokine levels in activated microglia Glia 2007 55 516 526 10.1002/glia.20478 17216595

[B35] Cross AK Woodroofe MN Chemokine modulation of matrix metalloproteinase and TIMP production in adult rat brain microglia and a human microglial cell line in vitro Glia 1999 28 183 189 10.1002/(SICI)1098-1136(199912)28:3<183::AID-GLIA2>3.0.CO;2-3 10559777

[B36] Kumnok J Ulrich R Wewetzer K Rohn K Hansmann F Baumgartner W Alldinger S Differential transcription of matrix-metalloproteinase genes in primary mouse astrocytes and microglia infected with Theiler's murine encephalomyeleitis virus J Neurovirol 2008 14 205 217 10.1080/13550280802008305 18569455 PMC7095224

